# Integrated analysis of the lncRNA/circRNA-miRNA-mRNA expression profiles reveals novel insights into potential mechanisms in response to root-knot nematodes in peanut

**DOI:** 10.1186/s12864-022-08470-3

**Published:** 2022-03-28

**Authors:** Ping Xu, Hui Li, Xiaohua Wang, Ge Zhao, Xiaofei Lu, Shengjie Dai, Xiaoyu Cui, Mei Yuan, Zhenning Liu

**Affiliations:** 1 College of Agriculture and Forestry Science, Linyi University, Middle of Shuangling Road, Lanshan District, Linyi, 26000 China; 2 Key Laboratory of Peanut Biology and Genetic Improvement, Ministry of Agriculture and Rural Affairs, Shandong Peanut Reasearch Instute, Qingdao, 266100 China

**Keywords:** Peanut, Root-knot nematode, Molecular mechanism, Competing endogenous RNA, Regulatory network

## Abstract

**Background:**

Peanut is the most essential oil and food crop globally due to its high oil and protein content. Root-knot nematode infects peanut roots, causing poor development and severely limiting peanut yields worldwide. The discovery of peanut genome identified a considerable number of genetic loci controlling the peanut root-knot nematode; however, the molecular mechanism of root-knot nematode remains unknown.

**Results:**

The heterogeneous response to root-knot nematode stress in peanut roots was identified using whole-transcriptome RNA-seq. A total of 430 mRNAs, 111 miRNAs, 4453 lncRNAs, and 123 circRNAs were found to have differential expression between infected and non-infected peanuts. The expression profiles of the lncRNA/circRNA-miRNA-mRNA network were developed to understand the potential pathways that lead to root-knot nematodes in peanut roots. During root-knot nematodes stress, a total of 10 lncRNAs, 4 circRNAs, 5 miRNAs, and 13 mRNAs can create competing endogenous RNA and participate in the oxidation–reduction process as well as other biological metabolism processes in peanuts. The findings will highlight the role of peanut ceRNAs in response to root-knot nematodes.

**Conclusion:**

The GO classification and KEGG pathway enrichment study of core regulatory networks revealed that ceRNAs are involved in oxidation–reduction, peroxidase activity, lignin synthesis in the xylem, and flavonoid synthesis. Overall, these findings may help researchers better understand the role of non-coding RNAs in response to root-knot nematodes.

**Supplementary Information:**

The online version contains supplementary material available at 10.1186/s12864-022-08470-3.

## Background

The root-knot nematode (Meloidogyne incognita) is one of the most dangerous plant-parasitic nematodes in the world [1]. Chemical insecticides, crop rotation, and host plant resistance were the primary means of controlling root-knot nematode for many years. Chemical insecticides (such as dibromochloropropane) have a crucial role in preventing and treating root-knot nematode disease, but they also generate significant environmental pollution [2]. Furthermore, due to the wide feeding range of worms in the kingdom Plantae, crop rotation has limited nematode control [3]. As a result, it is essential to develop crop resistance against root-knot nematode disease.

Peanut is a major oil crop, and roots nematode disease is one of the most dangerous diseases in peanut production areas worldwide [4]. One of the most efficient approaches to control root-knot nematode disease is to cultivate and plant disease-resistant peanut species. Root-knot nematode resistance in peanut cultivars is weak at the moment, and no effective peanut cultivars against root-knot nematode have been found so far [2]. The regulation mechanism of peanut against root-knot nematode is complex, and previous studies have identified several genetic loci [5]. However, the significance of RNAs in peanut root-knot nematode features remained unknown.

The condensation of ribonucleotides through phosphoric acid two ester bonds results in RNA. RNA is classified as messenger RNA (mRNA) or non-coding RNA (ncRNA) based on its structure and function [6]. Non-coding RNAs are classified as long non-coding RNAs (lncRNAs), circular RNAs (circRNAs), and tiny RNAs (miRNAs) based on their molecular weight [7, 8]. MiRNAs bind to mRNA via complete or incomplete base complementation and regulate target gene expression by impeding translation or directly causing the degradation of mRNA [9]. However, endogenous RNAs that contain the miRNA binding domain, such as lncRNA and circRNA, may alleviate miRNA repression on target genes (mRNA) [10]. Next-generation sequencing technology significantly aided the identification of lncRNAs, circRNAs, and miRNAs in plants [6, 8]. The ncRNA plays a crucial role in plant response to biotic and abiotic challenges such as diseases and pests, temperature, and drought based on gene regulation at the post-transcriptional stage [11]. In Arabidopsis, the miR472-RDR6 could suppress the pathway-modulated PAMP through post-transcriptional disease resistance control [12]. Overexpression of miR160a and miR398b in Oryza sativa increased resistance to Magnaporthe oryzae [13]. The expression of mi172a was hindered upon Aspergillus flavus infection in Arachis hypogaea [14]. In peanuts, a total of 347 circRNAs influencing flowering times were discovered [15]. In Arabidopsis, 1583 heat stress-specific circRNAs were discovered [16]. A total of 481 and 545 lncRNA demonstrated differential expression under dark and blue light treatments in Arabidopsis, respectively [17]. There were 1229 differently expressed heat-responsive lncRNAs in Chinese cabbage [18]. In the maize root, a total of 40 distinct expressed lncRNAs implicated in nitrogen uptake were discovered [19]. The competitive endogenous (ce) RNA hypothesis has gained traction as a putative stress-resistance regulation mechanism in plants. In maize [20], a miRNA-regulated network including 8834 mRNAs, 117 lncRNAs, and 77 miRNAs was built. A putative ceRNAs network containing 33 miRNA and 186 lncRNAs was built in tomato chilling resistance [8]. A ceRNA containing three lncRNAs (lncR9A, lncR117, and lncR616) as well as miR398 has been shown to improve winter wheat cold tolerance [21]. One miRNA (osamiR156aL + 1), two mRNAs, and 13 lncRNAs were discovered in the ceRNAs regulatory network, which enriched the putative regulation mechanism of glyphosate-tolerant [22].

In this study, cDNA and RNA libraries were created from nematode-caused root knots (treatment) and the same region portions of root tissues (control) in peanut (Huayu 22). A total of 430 mRNAs, 111 miRNAs, 4453 lncRNAs, and 123 circRNAs were differentially expressed between root-knot nematode-infected and uninfected peanut tissues. The ceRNAs network was built using the interaction of mRNAs, lncRNAs, circRNAs, and miRNAs. This network has the potential to improve understanding of mechanisms in response to root-knot nematodes and improve edible oil and edible plant tissues for humans and livestock.

## Materials and methods

### Plant materials and phenotype evaluation

Huayu22 peanut seed (The peanut cultivar was recognized by China's Shandong Crop Variety Approval Committee in March 2003, https://baike.so.com/doc/5049390-5276442.html) was cultivated in the planting pot in the greenhouse in Linyi, China (117.24°E, 34.22°N) from May of 2019 to May of 2020. The soil was sourced from an experimental region in Qingdao, China (120.41°E, 36.39°N), where the root-knot nematode was extensively burst. The root-knot nematode reproduced and grew in growing tomato plants. Tomato plants were used to propagate root-knot nematodes [8]. Wet sieving was used to collect the eggs from diseased tomato roots that had been sterilized. The peanut seed was sown after the eggs of root-knot nematodes were mixed with sandy soil. The trial's management adhered to standard breeding pot protocols. Peanuts flourish in a photoperiod of 16/8 light at 25 °C. Peanut samples with root-knot nematodes were collected and counted 21 days after germination (five-leaf stage planting). This study used three separate biological replicates samples gathered from different plants. The miRNAome and transcriptome analyses samples were maintained in a -80 °C freezer, and total RNA was extracted using the Trizol method [23, 24]. The SPSS software package (SPSS, statistics) was used to perform statistical analyses, and the one-way analysis of variance (ANOVA) was applied to determine statistical differences [24].

### The whole transcriptome sequencing and data analysis

Trizol reagent was used to extract total RNA from the peanut root. TruSeq Small RNA Sample Prep Kits were used to create the small RNA database (Illumina, San Diego, USA). Total RNA-seq provides a comprehensive whole-genome analysis. TruSeq RNA Sample Prep Kits were used to create the RNA libraries (miRNA and ribosome free strand-specific RNA sequencing library) after ligating the RNA 3' and 5' adapters (Illumina, San Diego, USA). The Illumina Hiseq 2 × 500 platforms sequence the prepared libraries (LC Science, Lianchuan Biotechnology Co., Ltd, Hangzhou, China). The Illumina procedure was used to obtain 2 × 150 bp paired-end reads and 150 bp single-end reads for mRNAs, lncRNAs, circRNAs, and miRNAs [8, 14].

High-quality reads with less than 5% missing nucleotides, sequence lengths more than 19 nt, and no continuous dimer nucleotides were chosen [8, 25]. Using TopHat version 2.1.1 software, the clean RNA-seq reads were mapped to the peanut (Arachis hypogaea L.) genome (http://peanutgr.fafu.edu.cn/index.php). After removing tRNA, scRNA, snoRNA, and rRNA from the GenBank non-coding RNA database, clean reads were assembled and merged to the final transcriptome using Cufflinksversion 2.2.1 and Cuffmergeversion 2.1.1 software, and the hairpin structures of miRNA precursors were predicted using Mireap v0.2 software (http://sourceforge.net/projects/mireap) [8]

### Differently expression mRNA, lncRNAs, circRNAsand miRNA identification

The differentially expressed mRNA between root-knot nematodes-infected peanut root and non-infected identical tissues was detected after the final transcriotome was generated using the stringent criterion of *P* < 0.01, FDR < 0.01, and |log2 (FPKM Treat/FPKM CK)|> 2 [24]. The differentially expressed lncRNAs were identified using the following criteria: greater than 200 bp, coding potential calculator < 0 and coding non coding index < 0, *P* < 0.01, Q < 0.01 and |log2 (FPKM Treat/FPKM CK)|> 2. Differentially expressed circRNAs were detected using the *P* < 0.01 and |log2 (SRPBM Treat/SRPBM _CK)|> 2 criteria [8]. To identify differentially expressed miRNAs, the following requirements were met: length from 18 to 25 nucleotides, *P* < 0.01 and |log2 (RPM Treat/ RPM _CK)|> 2 [14].

### Target prediction and the creation of a CE network

Co-expression, genetic co-location, and free energy building secondary structures between lncRNAs, circRNAs, miRNA, and mRNA were used to determine the target of ncRNAs. After sequence blasting and free energy calculations, the lncRNA and circRNA were identified as candidate target ncRNAs of mRNA [8]. The potential association between miRNAs and mRNAs was determined through miRTarBase (http://mirtarbase.mbc.nctu.edu.tw/),TargetScan (http://www.Targetscan.org/) and RNA22 (https://cm.Jefferson.edu/rna22/) [26]. The miRNA-mRNA network was built using Targetfinder software with the following criteria: one mismatch and base deletion are scored one; the match with G: U is scored 0.5, and the mismatch in the core region (from 2 to 13 nt) is scored one. This study defined the actual miRNA-mRNA regulation network as having a final alignment score of < 4. The miRNA-lncRNA/circRNA was constructed by software of Ssearch36 (36.3.6) of biomarker technology (http://www.biocloud.net/zhuanyongji) with criteria: the bulge is in the middle of mature miRNA; the mismatches of the base were less than 4, two consecutive mismatches of the base were deleted. The ceRNA network was built and visualized using Cytoscape V 3.6.1 software based on the given association.

### Enrichment analysis using GO and KEGG

The top GO R packages and KOBAS software were used to assess the biological functions of target ncRNA by GO classification and KEGG pathway enrichment analysis. GO annotations will be classified into three categories if the *p*-value is less below 0.01 (cellular component, molecular function, and biological process). Furthermore, with a false discovery rate (FDR) < 0.05, biological signal pathways of mRNAs and lncRNAs may be identified using the KEGG pathway database [26].

### RT-PCR quantitative analysis

Revert Aid First Strand cDNA Synthesis kit (Cat No: K1621, Thermo Scientific, Lithuania, EU) and Mir-XTM miRNA First-Stand Synthesis kit (Cat No: 638313, Takara Bio USA, Inc.) was used to generate total cDNA for the mRNA and miRNA expression profiles, respectively. The mRNA and miRNA primers are presented in Table S8. By using stem-loop quantitative RT-PCR (qRT-PCR) and qRT-PCR, researchers could determine the relative expression of miRNA and mRNA. On a RocheLightCycler 96RTPCR System (Roche, Germany), real-time qPCR was used to validate gene expression using 2 × SYBR® Green Supre-mix with the following thermal cycling conditions: 3 min at 95 °C, then 45 cycles of 5 s at 95 °C and 34 s at 60 °C. The melting curve was studied at temperatures ranging from 65 to 97 °C with a 1 °C per cycle increment and 4-s hold duration. The relative quantification of gene expression was measured using the two-dimensional computed tomography (CT) approach. MiRNA and mRNA expression studies were conducted using the SPSS software suite [24].

## Results

### Different peanut root morphology types caused by root-knot nematodes

**Fig. 1:**
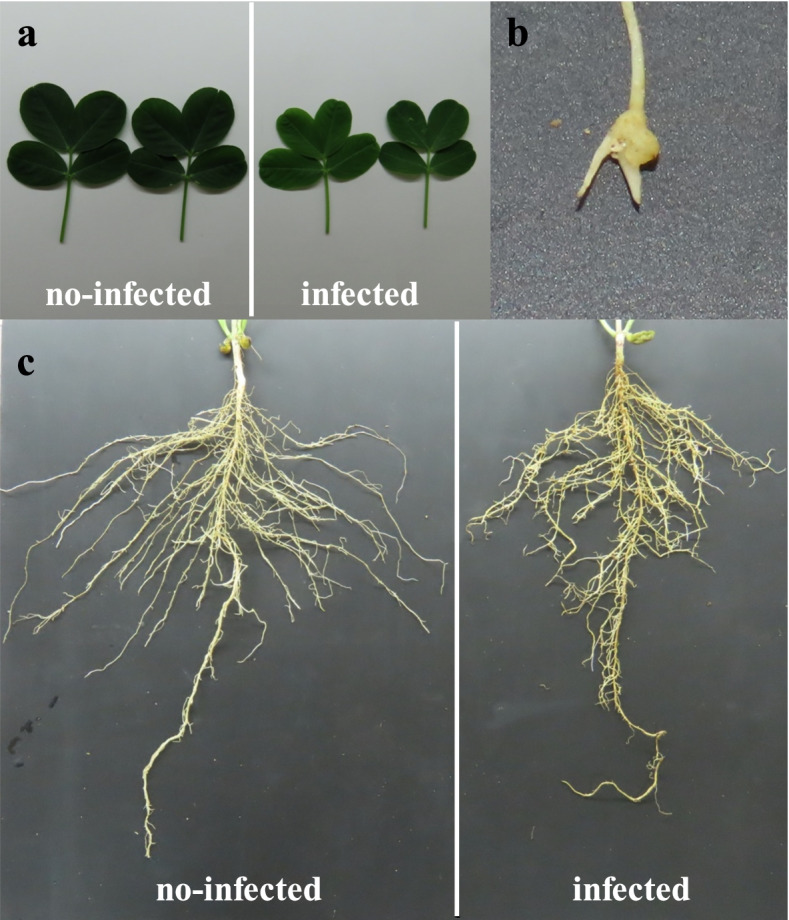
The infected and no-infected peanut (Huayu22) phenotype. **a **The different colors and size leaf between no-infected and infected peanut. **b** The peanut root-knot. **c **The root morphology types caused by root-knot nematodes

**Fig. 2:**
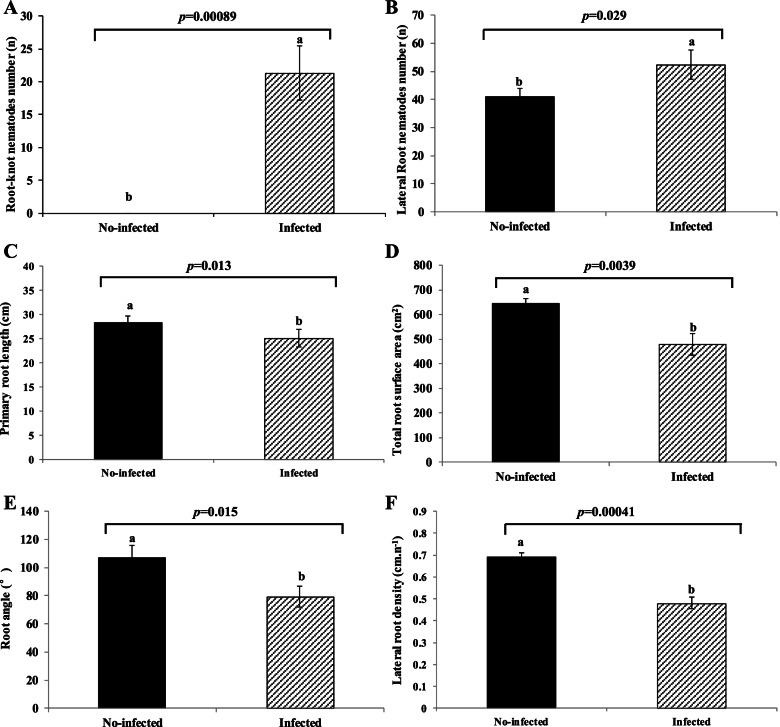
The no-infected and infected peanut root morphology traits caused by root-knot nematodes

The Huayu22 infected with the root-knot nematode showed a considerable increase in root node number (21.33 ± 4.416 vs. 0, *p* = 0.00089) as compared to the non-infected peanut (Figs. 1 and 2a, Table 1). Concurrently, root-knot nematodes may cause short primary root length (25.06 ± 1.79 vs. 28.26 ± 1.38, *p* = 0.0013) (Fig. 2c, Table 1), reduced gross root surface area (644.94 ± 21.53 vs. 478.36 ± 42.96, *p* = 0.0039) (Fig. 2d, Table 1), narrower root angle (79.26 ± 7.47 vs. 106.58 ± 8.94, (Figs. 1 and 2b, Table 1), less lateral root density (0.48 ± 0.026 vs. 0.69 ± 0.021, *p* = 0.00041) (Fig. 2e, Table 1) but more lateral root number (52.33 ± 5.13 vs. 41 ± 3,*p* = 0.029) in infected peanut (Figs. 1 and 2b, Table 1).

**Table 1:** The difference root morphology types between no-infected and infected peanut caused by root-knot nematodes

Traits	No-infected peanut	Infected peanut
Root-knot nematode number (n)	0	21.33 ± 4.16^**^
Primary root length (cm)	28.26 ± 1.38^**^	25.06 ± 1.79
Lateral root number (n)	41 ± 3	52.33 ± 5.13^*^
Total root surface area (cm^2^)	644.94 ± 21.53^**^	478.36 ± 42.96
Root angle (°)	106.58 ± 8.94^*^	79.26 ± 7.47
Lateral root density (cm/n)	0.6902 ± 0.0208^**^	0.4801 ± 0.0263

*P* < 0.01 is considered as highly significant and labeled as ^**^. *P* < 0.05 is considered as significant and labeled as ^*^

### The differential expression mRNA in response to peanut root-knot nematode

The quality score of the entire transcriptome sequencing is shown in Fig. S1. The highest base calling score from complete transcriptome sequencing was 36 (Sanger/Illumina 1.9 encoding). Infected and non-infected peanut rRNA had Rfam values ranging from 46.99% to 65.00%. Concurrently, the percentages of tRNA, snRNA, and snoRNA varied between 28.11 and 46.49%, 1.17 and 2.41%, and 0.95 and 1.98%, respectively. In this work, the length distribution of total sRNA counts was 24, implying that the sequencing and libraries were high quality.

**Fig. 3:**
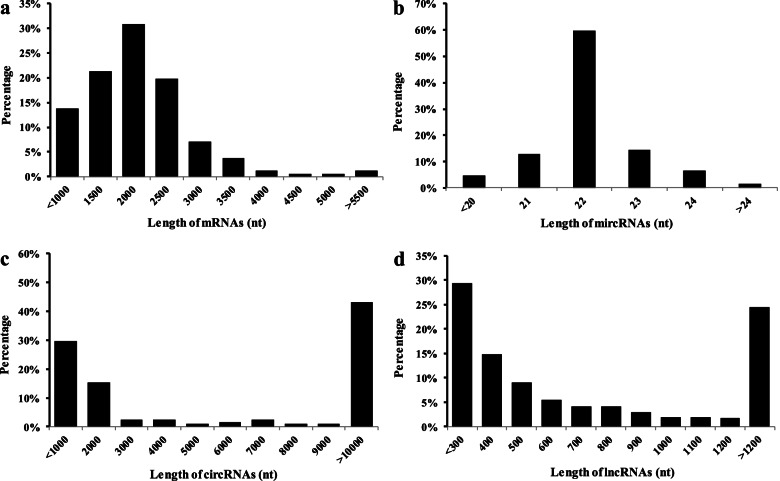
Sequence length distribution of differential expressed mRNAs, miRNAs, circRNAs and lncRNAs. **a **The length of differential expressed mRNAs. **b **The length of differential expressed miRNAs. **c** The length of differential expressed circRNAs. **d** The length of differential expressed lncRNAs

After studying the entire genome gene expression profile of peanuts in response to root-knot nematodes, researchers discovered a total of 430 differentially expressed mRNAs (427 up-regulated and 3 down-regulated) in response to root-knot nematodes (Fig. S3a, Table S1). The length of differentially expressed mRNA ranged from 200 to 12,283 base pairs (with an average of 1672 base pairs) (Fig. 3c, Table S1).

Differentially expressed genes were found on all 20 chromosomes. The number of differentially expressed mRNAs ranged from 11 on A02 chromosome to 35 on A01 and A03 chromosomes (with an average of 21.5). Sub-A genome (213) and sub-B genome (217) both have a similar number of differentially expressed genes (Fig. S2). Heat shock cognate protein, cytochrome P450, pathogenesis-related protein, peroxidase, and WRKY and MYB transcription factors were among the 329 differentially expressed genes (329 out of 430) that were found to be associated with stress adaptation (Table S1). Defense response, oxidation–reduction process, signal transduction, response to injury, nucleus, protein binding, and other GO annotations were among the top GO annotations of differentially expressed mRNAs (Table S1).

### The differential expression of miRNAs and their target genes

The expression patterns of miRNAs and target genes were discovered to demonstrate the involvement of miRNA in response to root-knot nematodes. The length of differentially expressed miRNA ranged from 19 to 25 nucleotides (on average, 21.75) (Fig. 3, Table S2). A total of 77 miRNAs were differentially expressed between root-knot nematode infected and non-infected cells. Furthermore, 35 of 77 genes were found to be up-regulated in exposure to root-knot nematodes (Fig. S3, Table S2).

**Fig. 4:**
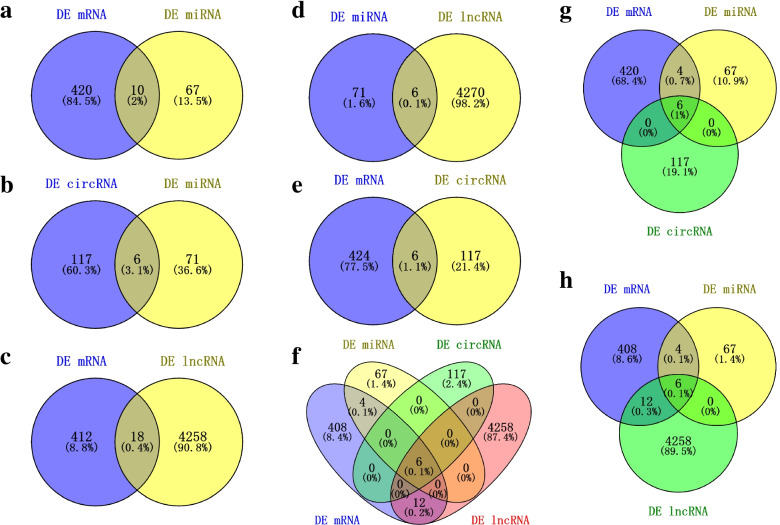
Expression analysis of root-knot nematodes-related mRNA and miRNAs using qRT-PCR and whole transcriptome resequencing. **a** The relative expression of key mRNA involved in the root-knot nematode stress ceRNAs network using qRT-PCR. **b** The expression of key mRNA involved in the root-knot nematode stress ceRNAs network using whole transcriptome resequencing. **c **The relative expression of key miRNA involved in the root-knot nematode stress ceRNAs network using qRT-PCR. **d** The expression of key miRNA involved in the root-knot nematode stress ceRNAs network using whole transcriptome resequencing

**Fig. 5:**
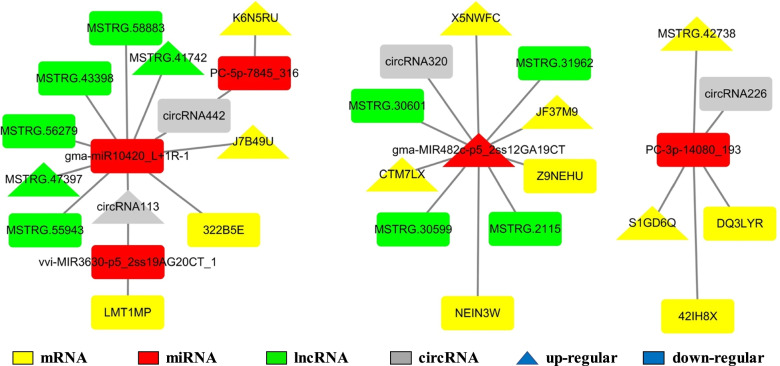
The number of differential expressed mRNAs, miRNAs, circRNAs, lncRNAs and the construction of regulatory networks. **a** The differential expressed miRNAs and the target mRNAs. **b** The differential expressed miRNAs and the target circRNAs. **c** The co-expressed differential expressed mRNAs and the lncRNAs. **d** The differential expressed miRNAs and the target lncRNAs. **e** The co-expressed differential expressed mRNAs and the circRNAs. **f** The ceRNAs regulatory network contains lncRNA/circRNA-miRNA-mRNA. **g** The mRNA-miRNAs-circRNAs regulator network. **h** The mRNA-miRNAs-lncRNAs regulator network

The target genes of those 77 miRNAs comprised 1771 mRNAs. In response to root-knot nematodes, 111 out of 1771 target genes were found to be differentially expressed (Table S3). Disease resistance-like protein DSC1, peroxidase, and WRKY transcription factor were among the 111 target genes annotated. Target genes were shown to be engaged in a variety of adversity response pathways, including plant-pathogen interaction, MAPK signaling pathway-plant, and starch and sucrose metabolism, according to GO and KEGG analysis (Table S3). The mRNAs-miRNAs regulatory networks contained miRNA (gma-MIR482c-p5 2ss12GA19CT) and mRNA (CTM7LX, JF37M9, NEIN3W, X5NWFC, and Z9NEHU) (Fig. 4); miRNA (PC-3p-30685 85) and mRNA (AJ79N4, NK4UTA, R13KY7, andXA3BQV); miRNA (PC-3p-14080 193) and mRNA (42IH8X, DQ3LYR, and S1GD6Q) were constructed (Figs. 5a, 4 and S4).

### CircRNAs acts as the sponge of miRNAs in response to peanut root-knot nematode

In response to peanut root-knot nematodes, a total of 123 differentially expressed circRNAs (60 up-regulated and 63 down-regulated) were discovered (Figs. S3c and S5). The number of circRNAs on each chromosome is irregularly distributed, ranging from 1 on A03 chromosome to 15 on A09 chromosome (with an average of 6.15) (Fig. S5). Out of 123 differentially expressed circRNAs, 6 (two up-regulated and four down-regulated) were predicted to bind to 7 miRNAs (with two up-regulated and five down-regulated). According to GO and KEGG analyses, the differentially expressed circRNAs are involved in defense response, response to oxidative stress, reaction to temperature stimuli, and so on (Table S4). The circRNAs-miRNAs regulatory channels were constructed by circRNAs (circRNA113 and circRNA442) and miRNA (gma-miR10420 L + 1R-1); circRNAs (circRNA226) and miRNA (PC-3p-14080 193); circRNAs (circRNA320) and miRNA (gma-MIR482c-p5 2ss12GA19CT); circRNAs (circRNA43) and miRNA (ptc-miR393a-3p) (Figs. 5b and S4).

### The role of lncRNAs played in regulatory ceRNA under root-knot nematode stress

By using a false discovery rate (FDR) of 0.05, 4439 significantly differentially expressed lncRNAs were discovered, of which 2904 differentially expressed lncRNAs were up-regulated, and 1535 differentially expressed lncRNAs were down-regulated (Fig. S3d, Table S5).

The length of differentially expressed lncRNAs ranged from 200 to 93,464 nucleotides (most lncRNAs were 200–300) (Fig. 3d). There were 13 differentially expressed lncRNAs (6 up-regulated and 7 down-regulated) bind to 6 miRNAs (with 3 up-regulated and 3 down-regulated). The lncRNAs-miRNAs regulator interactions were constructed by lncRNAs (MSTRG.12823, MSTRG. 17,002, MSTRG.33245 and MSTRG.42738) and miRNA (PC-3p-14080 193 and PC-3p-30685 85); lncRNAs (MSTRG.2115, MSTRG.30601, MSTRG.30599 and MSTRG.31962) and miRNA (gma-MIR482c-p5 2ss12GA19CT); lncRNAs (MSTRG.3150 and MSTRG.37521) and miRNA (mtr-miR319a-3p R + 1) (Figs. 5d and S3).

### The regulatory ceRNA network of lncRNA/circRNA-miRNA-mRNA in response to root-knot nematode stress

**Fig. 6:**
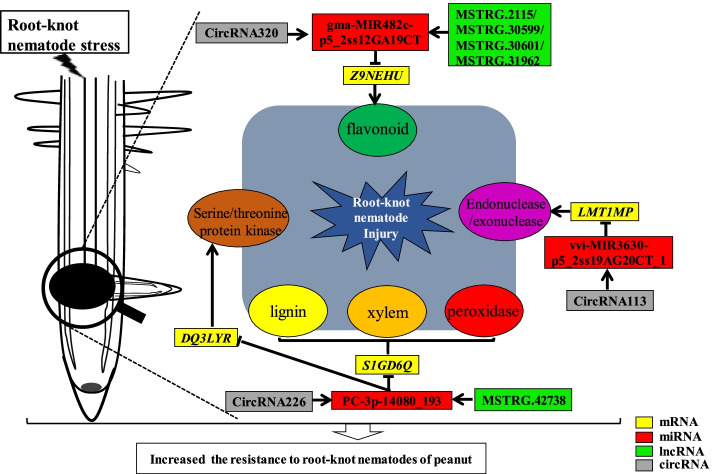
The regulatory ceRNA network of lncRNA/circRNA-miRNA-mRNA in response to root-knot nematode stress

**Fig. 7:**
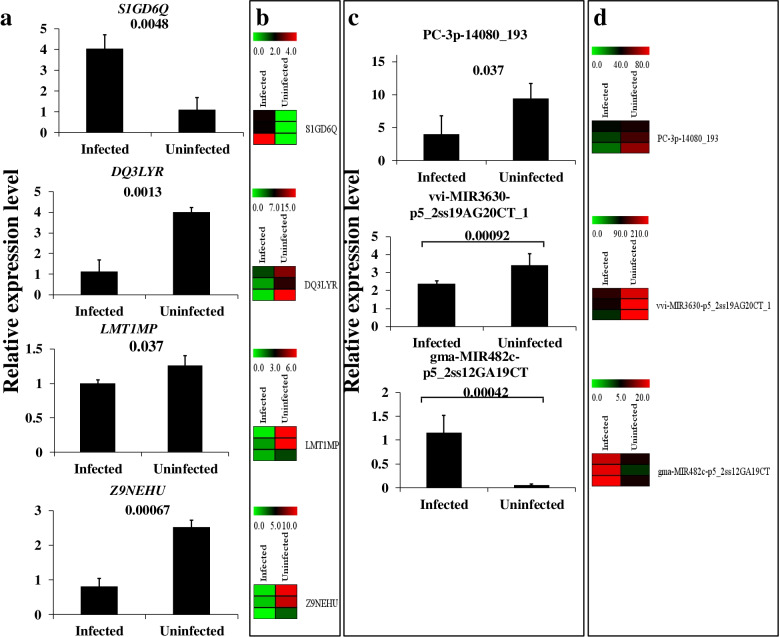
Regulator network model of the miRNAs-mediated lncRNA, circRNA and mRNA involved in root-knot nematode stress

Differentially expressed lncRNA, circRNA, mRNA, and miRNA were discovered (Figs. 3 and S3, Tables S1-S7). Reconstruction of the critical lncRNA/circRNA-miRNA-mRNA competitive endogenous RNA tetraploid sub-network linked to root-knot nematode stress response (Figs. 5 and S3, Tables S3, S6, and S7). The first sub-network included six lncRNAs (MSTRG.41742, MSTRG.43398, MSTRG.47397, MSTRG.55943, MSTRG.56279, and MSTRG.58883), two circRNAs (circRNA113 and circRNA442), three miRNAs (gma-miR10420 L + 1R-1, vvi-MIR3630-p5 2ss19 (LMT1MP, J7B49U, 322B5E, and K6N5RU). In response to the peanut root-knot nematode, two lncRNAs, one circRNA, and two mRNA were shown to be up-regulated. Meanwhile, four, one, three, and two lncRNAs, circRNAs, miRNAs, and mRNAs were shown to be down-regulated under root-knot nematode stress, correspondingly. Four lncRNAs (all down-regulated), one circRNA (down-regulated), one miRNA (up-regulated), and five mRNAs make up the second sub-network (three up-regulated and two down-regulated). One down-regulated circRNA, one up-regulated miRNA, and four mRNAs (two up-regulated and two down-regulated) were used to create the final sub-network (Figs. 4, 6 and S4). According to GO and KEGG analyses, the RNAs in the ceRNA network were engaged in peroxidase activity, lignin biosynthetic process, and oxidation–reduction process (Figs. 7 and S6).

## Discussion

Root-knot nematodes are one of the most destructive agricultural pests globally [1]. After being infected with root-knot nematodes, several physiological and biochemical changes occurred, including root tissue necrosis, decreased peanut root activity, and reduced peanut photosynthetic and respiratory intensity [27]. However, there was limited research on the role of peanut root morphology. The peanuts grown in the presence of root-knot worms had a shorter primary root length, a minor total root surface area, a narrower root angle, and a lower lateral root density but a higher lateral root number than those grown in the absence of root-knot nematodes (Figs. 1 and 2). The peanut root produced a root-knot after being infected with root-knot nematodes and did not extend. The most important organs for receiving nutrients and water are the roots. Root-knot hinders peanuts from absorbing nutrients from their environment. After being infected by the root-knot nematode, peanut roots generated additional lateral roots to absorb more nutrients [28]. Root-knot nematodes, on the other hand, have limited movement. As a result, the distribution of the root-knot nematode in the soil is critical for peanut disease. We used tomato infection to breed root-knot nematodes and incubate them quickly. The nematode was then extracted and evenly distributed throughout the soil.

A series of broad host plant gene regulations was generated when plants were infected with root-knot nematodes. Recent investigations have discovered several resistance genes (Mi, Ma, rhg1, TIR-NBS-LRR gene) in tomato, plum, soybean, and peanut [5, 29–31]. However, it remained unclear what role RNAs had in the regulation mechanism of peanut against root-knot nematode. In this study, 430 differentially expressed mRNAs, 77 differentially expressed miRNAs, 123 differentially expressedcircRNAs, and 4439 differentially expressed lncRNAs were implicated in the root-knot nematode regulatory mechanism investigated (Fig. S3, Tables S1-S5). Meanwhile, under soybean cyst nematode stress, the number of differentially expressed genes in resistant and susceptible genotypes of Glycine soja was 2290 and 555, respectively [32]. In peanuts, there were 5595 differentially expressed mRNA responses to nematode infection. In diverse peanut genotypes, 3178 genes out of 5595 formed three co-expression gene clusters, independent of resistance and susceptibility types [5]. According to the top enrich GO analysis, the constitutively differentially expressed genes involved in cell proliferation and stress resistance, such as cell division, DNA replication initiation, mitosis, stress response, salt stress response, and water deprivation, implying that resistant peanut root genotypes had greater growth vitality than susceptible peanut root genotypes under nematode infection [5]. Defense reaction, including defense response to bacteria, oomycetes, virus, a gram-negative bacterium, and fungus, and cell proliferation process, including DNA binding transcription factor activity, were both examined in our studies (Figs. 7 and S6). In conclusion, several lncRNA, circRNA, miRNA, and mRNA were found to be differentially expressed in response to peanut root-knot nematode stress, implying that RNAs were important in root morphological changes and regulation mechanisms in peanut against root-knot nematodes.

The competing endogenous RNA model (Fig. 5) associated with root-knot nematode stress response was reconstructed using miRNA-mediated differentially expressed lncRNA, circRNA, and mRNA. Peroxidase activity, the lignin, and the flavonoid biosynthesis process were all affected by the target miRNA-mediated regulatory RNAs that were differentially expressed in the stress-resistant biological processes (Figs. 7 and S6, Tables S1-S7). Increased miRNA (gma-MIR482c-p5 2ss12GA19CT) down-regulated the mediator of RNA polymerase II transcription subunit 15-like isoform X1 (NEIN3W) () when peanut was exposed to root-knot nematode stress, according to the ceRNA network. To lessen the inhibition of mRNA by miRNA, one circRNA (circRNA320) and four lncRNAs (MSTRG.2115, MSTRG.30599, MSTRG.30601, and MSTRG.31962) established a competitive endogenous RNA model to maintain the growth of peanut roots. Meanwhile, to sustain the peanut development under nematode stress, the expression of the JF37M9 gene (CLK4-associating serine/arginine-rich protein) was elevated. Pre-mRNA splicing regulator—serine/arginine-rich protein plays an essential function in plant response to high-temperature stress [33]. Cold-responsive protein kinase 1-like isoform X4 (DQ3LYR) and G-type lectin S-receptor-like serine/threonine-protein kinase At5g35370 isoform X1 gene (42IH8X) activities were both suppressed by another regulatory network. Plant tolerance to salt stress is positively regulated by the G-type lectin S-receptor-like serine/threonine-protein kinase [34]. The synthesis of peroxidase was enhanced (S1GD6Q) using circRNA (circRNA226) and lncRNA (MSTRG.42738), resulting in the scavenging of free radicals and the reduction of damage to peanut roots (Figs. 7 and S4).

Together, the integrated analysis of the lncRNA/circRNA-miRNA-mRNA expression profiles may likely reveal the complex ceRNAs regulatory network and mechanism that operates during the stressful condition of peanut root-infested with nematodes.

## Conclusion

This study identified 430 mRNAs, 77 miRNAs, 4439 lncRNAs, and 123 circRNAs to have differential expression between infected and non-infected peanuts using whole transcriptome RNA-seq. In peanut, a total of 10 lncRNAs, 4 circRNAs, 5 miRNAs, and 13 mRNAs can consistently regulate mRNA production during root-knot nematode stress by creating competing endogenous RNA and participating in the oxidation–reduction process as well as other biological metabolism pathways. The results obtained will give insight into the importance of ceRNAs in peanut response to root-knot nematodes.

## Supplementary Information

**Additional file 1: Supplementary Figure 1.** The transcriptome assembly quality control. a, The base quality of sequence. b, The pie chart for Rfam sequence category. c, the Biological Replicate quality control. d, The length distribution of counts of total sRNAs in this study.

**Additional file 2:** **Supplementary Figure 2.** The physical location of differentially expressed mRNAs in response to peanut root-knot nematode.

**Additional file 3:** **Supplementary Figure 3.** The regulation of differential expressed mRNAs, miRNAs, circRNAs and lncRNAs.

**Additional file 4: Supplementary Figure 4.** The construction of regulatory networks contains lncRNA, circRNA, miRNA and mRNA in peanut response to root -knot nematode.

**Additional file 5:** **Supplementary Figure 5.** The physical location of differentially expressed circRNAs in response to peanut root-knot nematode.

**Additional file 6:** **Supplementary Figure 6.** The differential expressed mRNAs, top GO annotation and KEGG analysis. a. The volcanic map of differential expressed mRNAs. b. The top GO annotation of differential expressed mRNAs. c. The KEGG analysis of differential expressed mRNAs.

**Additional file 7: Supplementary Table 1.** The differential expression mRNAs between infected and no-infected peanut.

**Additional file 8:** **Supplementary Table 2.** The differential expression miRNAs between infected and no-infected peanut.

**Additional file 9:** **Supplementary Table 3.** The differential expressed miRNAs and their target mRNAs.

**Additional file 10: Supplementary Table 4.** The differential expression circRNAs between infected and no-infected peanut.

**Additional file 11:** **Supplementary Table 5.** The differential expression lncRNAs between infected and no-infected peanut.

**Additional file 12:** **Supplementary Table 6.** The differential expressed miRNAs and their target circRNAs.

**Additional file 13: Supplementary Table 7.** The differential expressed mRNAs and co-expression lncRNAs.

**Additional file 14: Supplementary Table 8.** The key primers of mRNA and miRNA used forquantitative RT-PCR analysis.

## Data Availability

Data and materials were provided it in the Supplementary Tables 1–7.

## Abbreviations

ceRNAs: Competing endogenous RNA
P: Phosphorus
ncRNA: non-coding RNA
DE: Differently expression

## References

[1] Abad P, Gouzy J, Aury J.M, Castagnone-Sereno P, Danchin E.G.J, Deleury E, Perfus-Barbeoch L, Anthouard V, Artiguenave F, Blok VC (2008). Genome sequence of the metazoan plant-parasitic nematode *Meloidogyne incognita*. Nat. Biotechnol..

[2] Wang H, Shi YM, Ren Y, Li SL, Jiao K, Yuan M, Li HJ (2008). Development of SSR markers for root-knot nematode resistance in peanut. J Peanut Sci.

[3] Dong W, Holbrook CC, Timper P, Brenneman TB, Mullinix BG (2007). Comparison of methods for assessing resistance to Meloidogyne arenaria in peanut. J Nematol.

[4] Zhuang W, Chen H, Yang M, Wang J, Pandey MK, Zhang C, Chang WC, Zhang L, Zhang X, Tang R (2019). The genome of cultivated peanut provides insight into legume karyotypes polyploid evolution and crop domestication. Nat. Genet.

[5] Josh C, Ye C, Larissa AG, Thiago M, David B, Soraya LB, Patricia TC, Corley H, Peggy OA (2017). Gene expression profiling describes the genetic regulation of meloidogyne arenaria resistance in *Arachis hypogaea* and reveals a candidate gene for resistance. Sci Rep.

[6] Wang J, Yu W, Yang Y, Li X, Chen T, Liu T, Ma N, Yang X, Liu R, Zhang B (2015). Genome-wide analysis of tomato long non-coding RNAs and identification as endogenous target mimic for microRNA in response to *TYLCV* infection. Sci Rep.

[7] Schneider T, Bindereif A (2017). Circular rnas coding or noncoding?. Cell Res.

[8] Wang Y, Gao L, Zhu B, Zhu H, Luo Y, Wang Q, Zuo J (2018). Integrative analysis of long non-coding RNA acting as ceRNAs involved in chilling injury in tomato fruit. Gene..

[9] Fabian MR, Sundermeier TR, Sonenberg N (2010). Understanding how miRNAs post-transcriptionally regulate gene expression. Prog Mol Subcell Biol.

[10] Lee S, Lee W, Ren S, Han K. A Method for Constructing an Integrative Network of Competing Endogenous RNAs. Intelligent Computing Theories and Application. 2021:407–20.

[11] Khraiwesh B, Zhu J, Zhu J (2012). Role of miRNAs and siRNAs in biotic and abiotic stress responses of plants. Biochim Biophys Acta-biomembranes.

[12] Boccara M, Sarazin A, Thiébeauld O, Jay F, Voinnet O, Navarro L, Colot V (2014). The Arabidopsis miR472-RDR6 silencing pathway modulates PAMP-and effector-triggered immunity through the post-transcriptional control of disease resistance genes. PLoS Pathog.

[13] Li Y, Lu YG, Shi Y, Wu L, Xu YJ, Huang F, Guo XY, Zhang Y, Fan J (2014). Multiple rice microRNAs are involved in immunity against the blast fungus *Magnaporthe oryzae*. Plant Physiol.

[14] Zhao C, Li T, Zhao Y, Zhang B, Wang X (2020). Integrated small RNA and mRNA expression profiles reveal miRNAs and their target genes in response to *Aspergillus flavus* growth in peanut seeds. BMC Plant Biol.

[15] Zhang X, Ma X, Ning L, Li Z, Yin D (2019). Genome-wide identification of circular RNAs in peanut (*Arachis hypogaea* L.). BMC Genomics..

[16] Pan T, Sun X, Liu Y, Li H, Deng G, Lin H, Wang S (2018). Correction to heat stress alters genome-wide profiles of circular RNAs in *Arabidopsis*. Plant Mol Biol.

[17] Sun Z, Huang K, Han Z, Wang P, Fang Y (2020). Genome-wide identification of *Arabidopsis* long noncoding RNAs in response to the blue light. Sci Rep.

[18] Song X, Hu J, Wu T, Yang Q, Feng X, Li H, Feng S, Cui C, Yu Y, Zhou R (2021). Comparative analysis of long noncoding RNAs in angiosperms and characterization of long noncoding RNAs in response to heat stress in Chinese cabbage. Hortic Res.

[19] Ma P, Zhang X, Luo B, Chen Z, He X, Zhang H, Li B, Liu D, Wu L, Gao S (2021). Transcriptomic and genome-wide association study reveal long noncoding RNAs responding to nitrogen deficiency in maize. BMC Plant Biol.

[20] Fan C, Hao Z, Yan J, Li G (2015). Genome-wide identification and functional analysis of lincRNAs acting as miRNA targets or decoys in maize. BMC Genomics.

[21] Lu Q, Guo F, Xu Q, Jing C (2020). LncRNA improves cold resistance of winter wheat by interacting with miR398. Funct Plant Biol.

[22] Zhai R, Ye S, Zhu G, Lu Y, Ye J, Yu F, Chu Q, Zhang X (2020). Identification and integrated analysis of glyphosate stress-responsive microRNAs lncRNAs and mRNAs in rice using genome-wide high-throughput sequencing. BMC Genomics.

[23] Xu P, Lv Z, Zhang X, Wang X, Pu Y, Wang H, Yi B, Wen J, Ma C, Tu J (2014). Identification of molecular markers linked to trilocular gene (*mc1*) in *Brassica juncea* L. Mol Breeding.

[24] Wang X, Chen Y, Thomas CL, Ding G, Xu P, Shi D, Grandke F, Jin K, Cai H, Xu F (2017). Genetic variants associated with root system architecture of oilseed rape under contrasting phosphate availabilities through genome –wide analyses. DNA Res.

[25] Chen H, Yang Q, Chen K, Zhao S, Zhang C, Pan R, Cai T, Deng Y, Wang X, Chen Y (2019). Integrated microRNA and transcriptome profiling reveals a miRNA-mediated regulatory network of embryo abortion under calcium deficiency in peanut (*Arachis hypogaea* L). BMC Genomics.

[26] Li D, Chen W, Luo L, Wang Y, Shang J, Zhang Y, Chen G, Li S (2017). Prospective lncRNA-miRNA-mRNA regulatory network of long non-coding RNA LINC00968 in non-small cell lung cancer A549 cells: a miRNA microarray and bioinformatics investigation. Int J Mol Med.

[27] Pandey RK (2020). Physiological and biochemical changes in susceptible and resistant rice cultivars induced by root-knot nematode. Meloidogyne Graminicola Indian Phytopathol.

[28] Elhady A, Hallmann J, Heuer H (2020). Symbiosis of soybean with nitrogen fixing bacteria affected by root lesion nematodes in a density-dependent manner. Sci Rep.

[29] Milligan SB, Bodeau J, Yaghoobi J, Kaloshian I, Zabel P, Williamson VM (1998). The root knot nematode resistance gene *Mi* from tomato is a member of the leucine zipper nucleotide binding leucine-rich repeat family of plant genes. Plant Cell.

[30] Claverie M, Dirlewanger E, Bosselut N, Ghelder CV, Voisin R, Kleinhentz M, Lafargue B, Abad P, Rosso MN, Chalhoub B, Esmenjaud D (2011). The Ma gene for complete-spectrum resistance to Meloidogyne species in Prunus is a TNL with a huge repeated C-terminal post-LRR region. Plant Physiol.

[31] CookD E, Lee T.G, Guo X, Melito S, Wang K, Bayless A.M, Wang J, Hughes T.J, Willis D.K, Clemente TE (2012). Copy number variation of multiple genes at *Rhg1* mediates nematode resistance in soybean. Science..

[32] Zhang H, Kjemtrup-Lovelace S, Li C, Luo Y, Chen L.P, Song B.H (2017). Comparative RNA-Seq analysis uncovers a complex regulatory network for soybean cyst nematode resistance in wild soybean (*Glycine soja*). Sci. Rep..

[33] Ling Y, Mahfouz MM, Zhou S (2021). Pre-mRNA alternative splicing as a modulator for heat stress response in plants. Trends Plant Sci.

[34] Sun X, Yu Q, Tang L, Ji W, Bai X, Cai H, Liu X, Ding X, Zhu Y (2013). GsSRK, a G-type lectin S-receptor-like serine/threonine protein kinase, is a positive regulator of plant tolerance to salt stress. J Plant Physiol.

