# Supplementary material for: Integrated analysis of the lncRNA/circRNA-miRNA-mRNA expression profiles reveals novel insights into potential mechanisms in response to root-knot nematodes in peanut
Source: BMC Genomics. 2022 Mar 28;23:239. doi: 10.1186/s12864-022-08470-3 (PMC8962500; doi:10.1186/s12864-022-08470-3)
Supplement: Supplementary file 1 — Additional file 1: Supplementary Figure 1. The transcriptome assembly quality control. a, The base quality of sequence. b, The pie chart for Rfam sequence category. c, the Biological Replicate quality control. d, The length distribution of counts of total sRNAs in this study. [file 12864_2022_8470_MOESM1_ESM.docx]

**Title: Integrated analysis of the lncRNA/circRNA-miRNA-mRNA expression profiles reveals novel insights into potential mechanisms in response to root-knot nematodes in peanut**

**Supplemantary Figure 1.** The transcriptome assembly quality control. a, The base quality of sequence. b, The pie chart for Rfam sequence category. c, the Biological Replicate quality control. d, The length distribution of counts of total sRNAs in this study.
