# Supplementary material for: Integrated analysis of the lncRNA/circRNA-miRNA-mRNA expression profiles reveals novel insights into potential mechanisms in response to root-knot nematodes in peanut
Source: BMC Genomics. 2022 Mar 28;23:239. doi: 10.1186/s12864-022-08470-3 (PMC8962500; doi:10.1186/s12864-022-08470-3)
Supplement: Supplementary file 3 — Additional file 3: Supplementary Figure 3. The regulation of differential expressed mRNAs, miRNAs, circRNAs and lncRNAs. [file 12864_2022_8470_MOESM3_ESM.docx]

**Title: Integrated analysis of the lncRNA/circRNA-miRNA-mRNA expression profiles reveals novel insights into potential mechanisms in response to root-knot nematodes in peanut**

**Supplemantary Figure 3.** The regulation of differential expressed mRNAs, miRNAs, circRNAs and lncRNAs.
