# Supplementary material for: Integrated analysis of the lncRNA/circRNA-miRNA-mRNA expression profiles reveals novel insights into potential mechanisms in response to root-knot nematodes in peanut
Source: BMC Genomics. 2022 Mar 28;23:239. doi: 10.1186/s12864-022-08470-3 (PMC8962500; doi:10.1186/s12864-022-08470-3)
Supplement: Supplementary file 4 — Additional file 4: Supplementary Figure 4. The construction of regulatory networks contains lncRNA, circRNA, miRNA and mRNA in peanut response to root -knot nematode. [file 12864_2022_8470_MOESM4_ESM.pptx]

## Slide 1
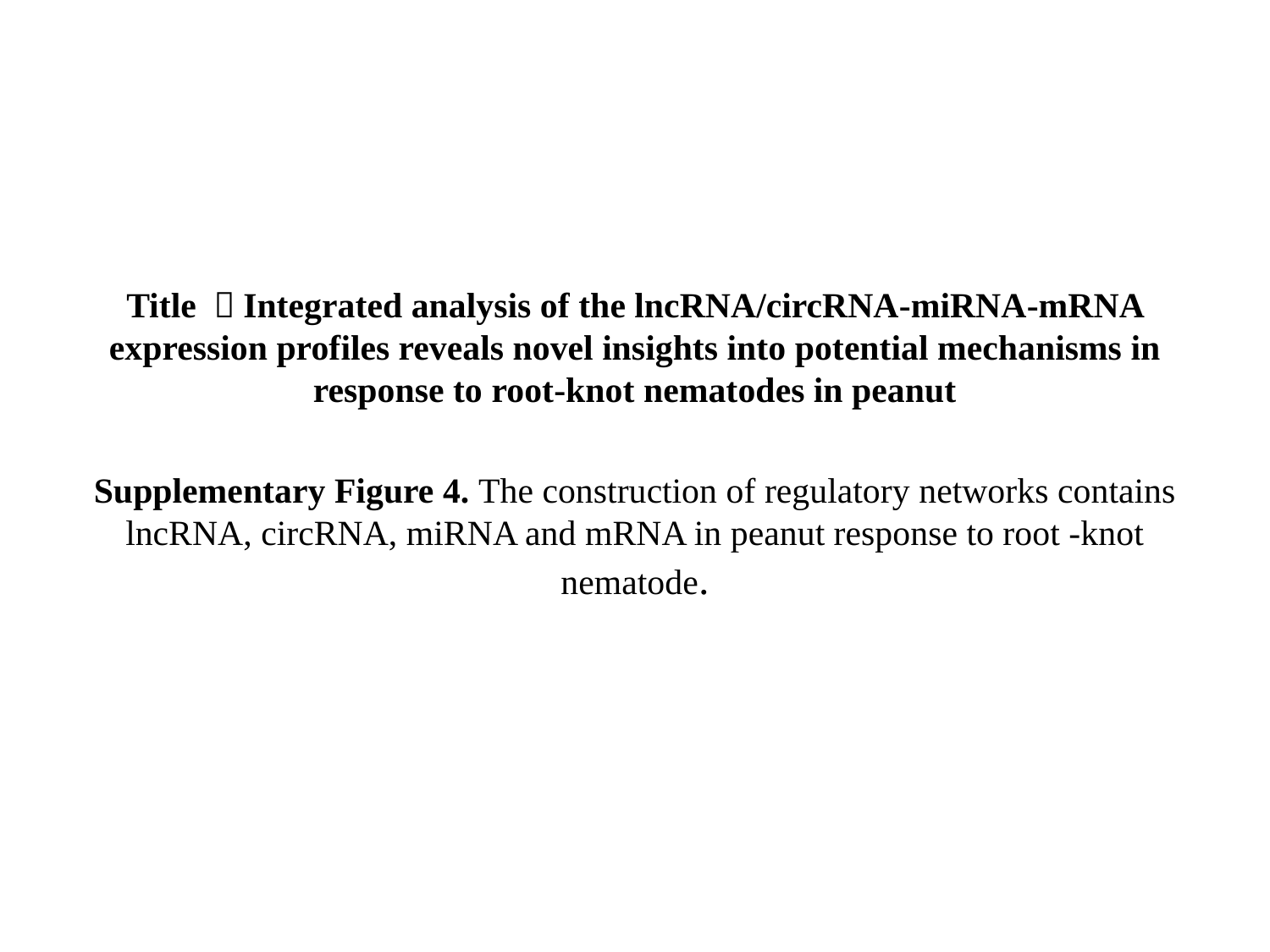

Title ：Integrated analysis of the lncRNA/circRNA-miRNA-mRNA expression profiles reveals novel insights into potential mechanisms in response to root-knot nematodes in peanut
Supplementary Figure 4. The construction of regulatory networks contains lncRNA, circRNA, miRNA and mRNA in peanut response to root -knot nematode.

## Slide 2
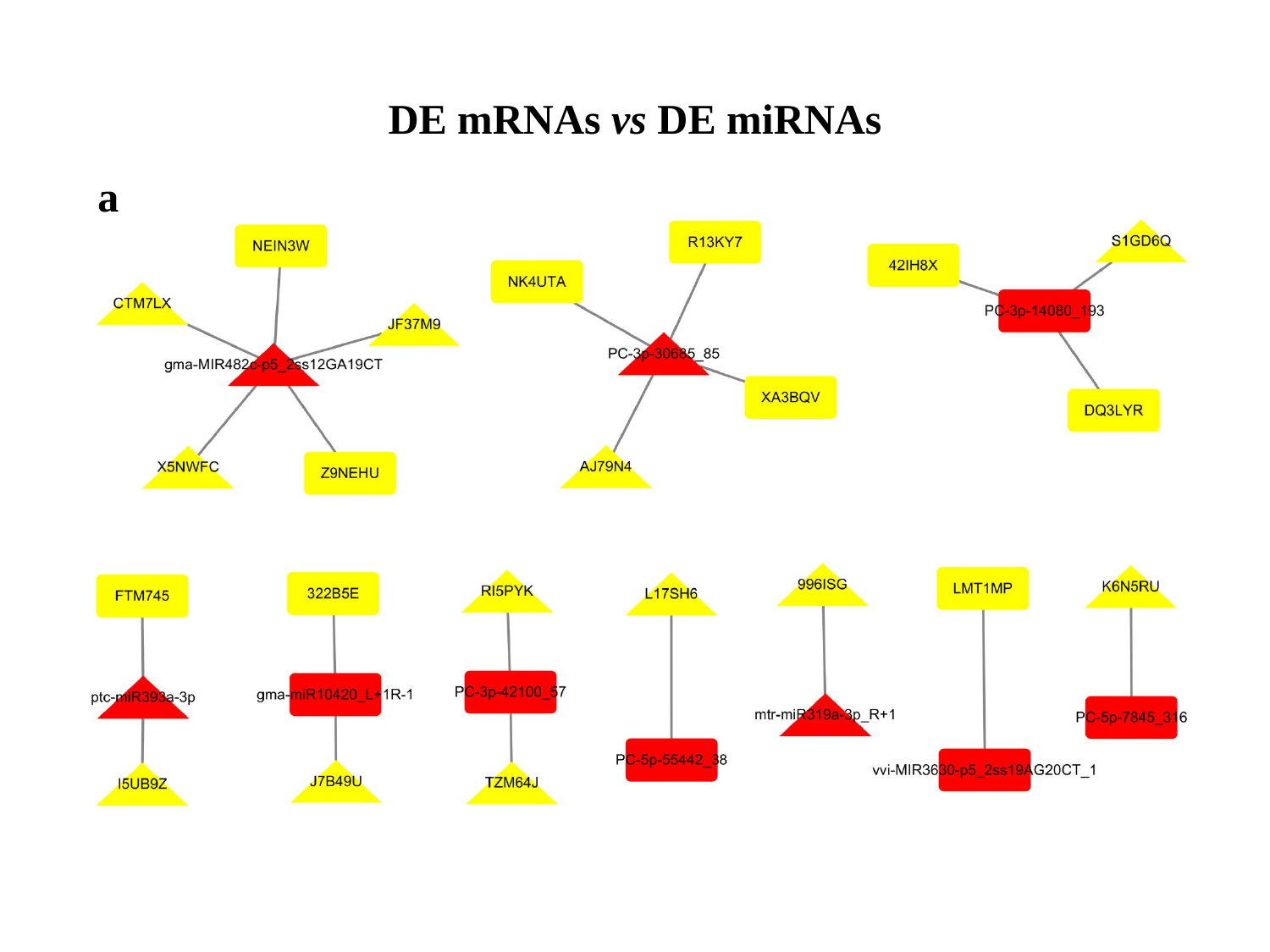

# DE mRNAs vs DE miRNAs
a

## Slide 3
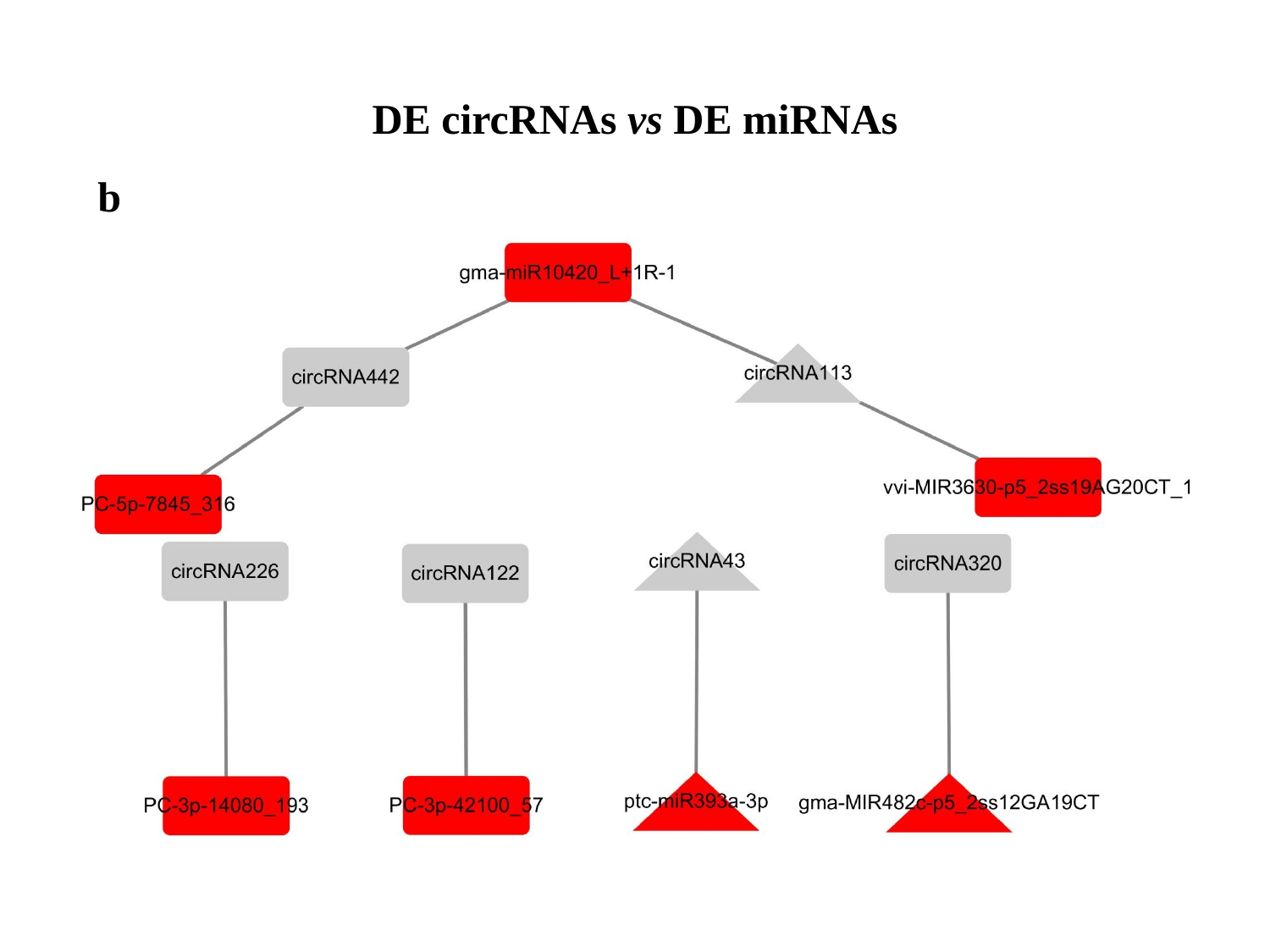

# DE circRNAs vs DE miRNAs
b

## Slide 4
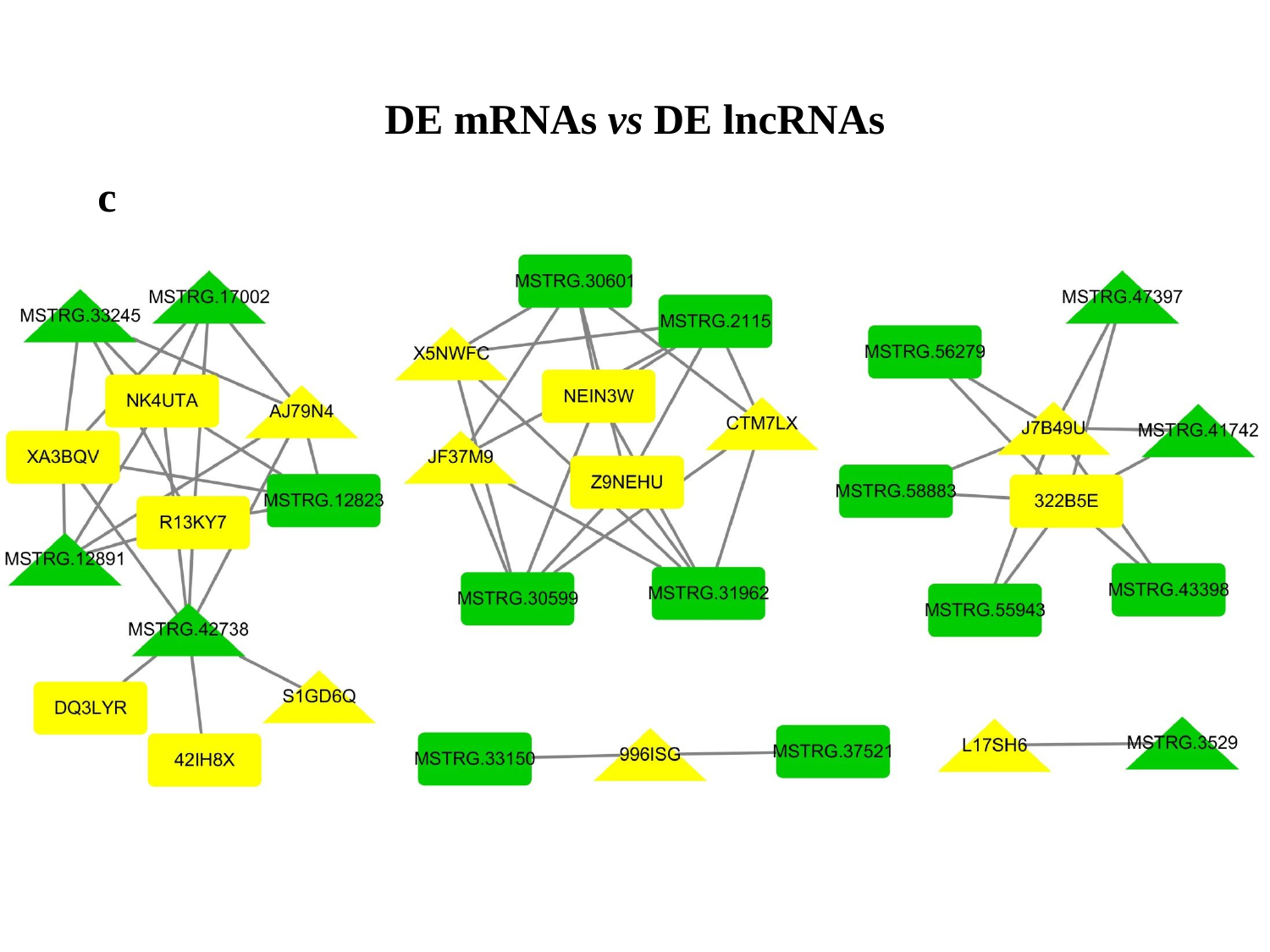

DE mRNAs vs DE lncRNAs
c

## Slide 5
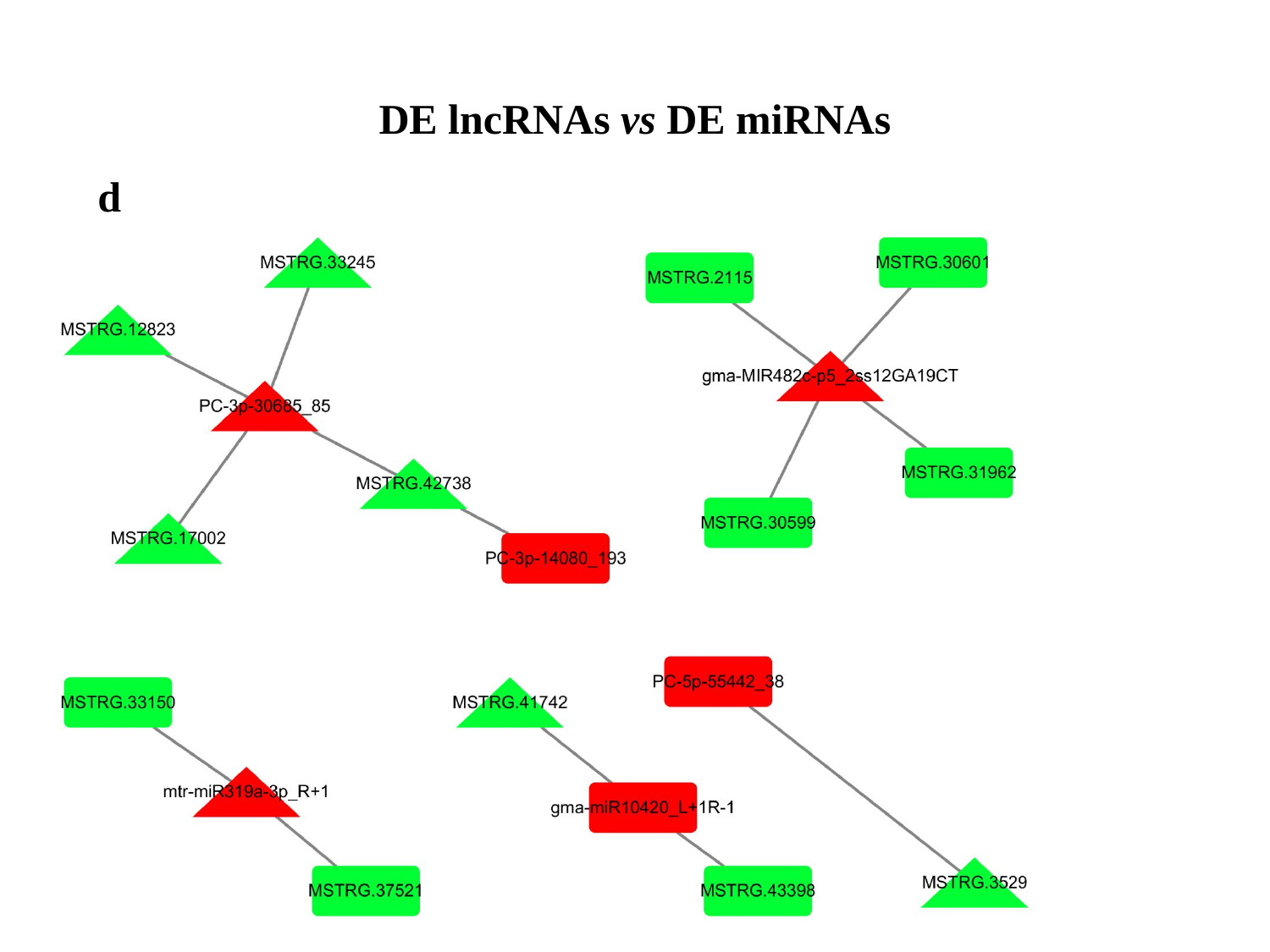

# DE lncRNAs vs DE miRNAs
d

## Slide 6
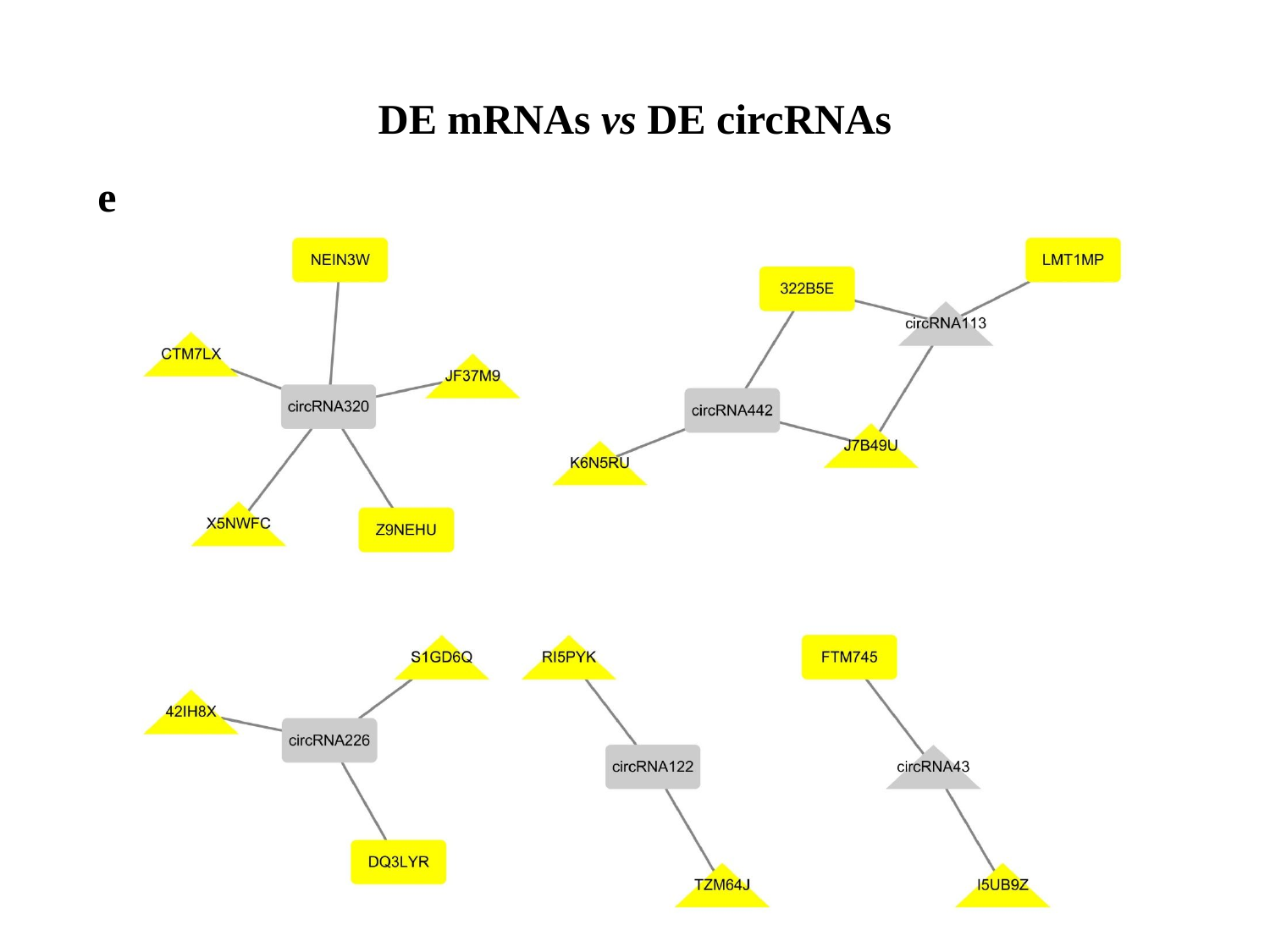

# DE mRNAs vs DE circRNAs
e

## Slide 7
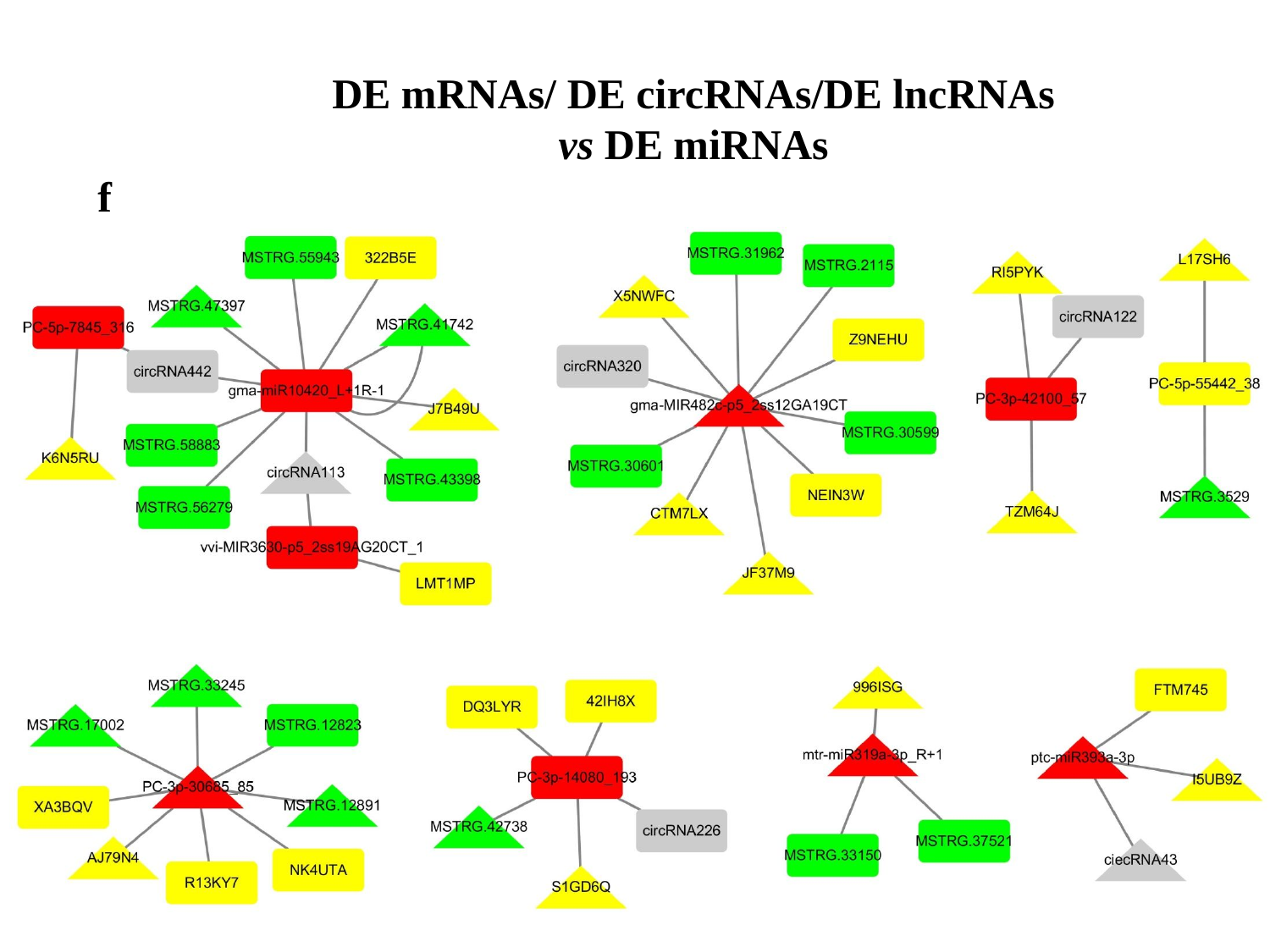

# DE mRNAs/ DE circRNAs/DE lncRNAs vs DE miRNAs
f

## Slide 8
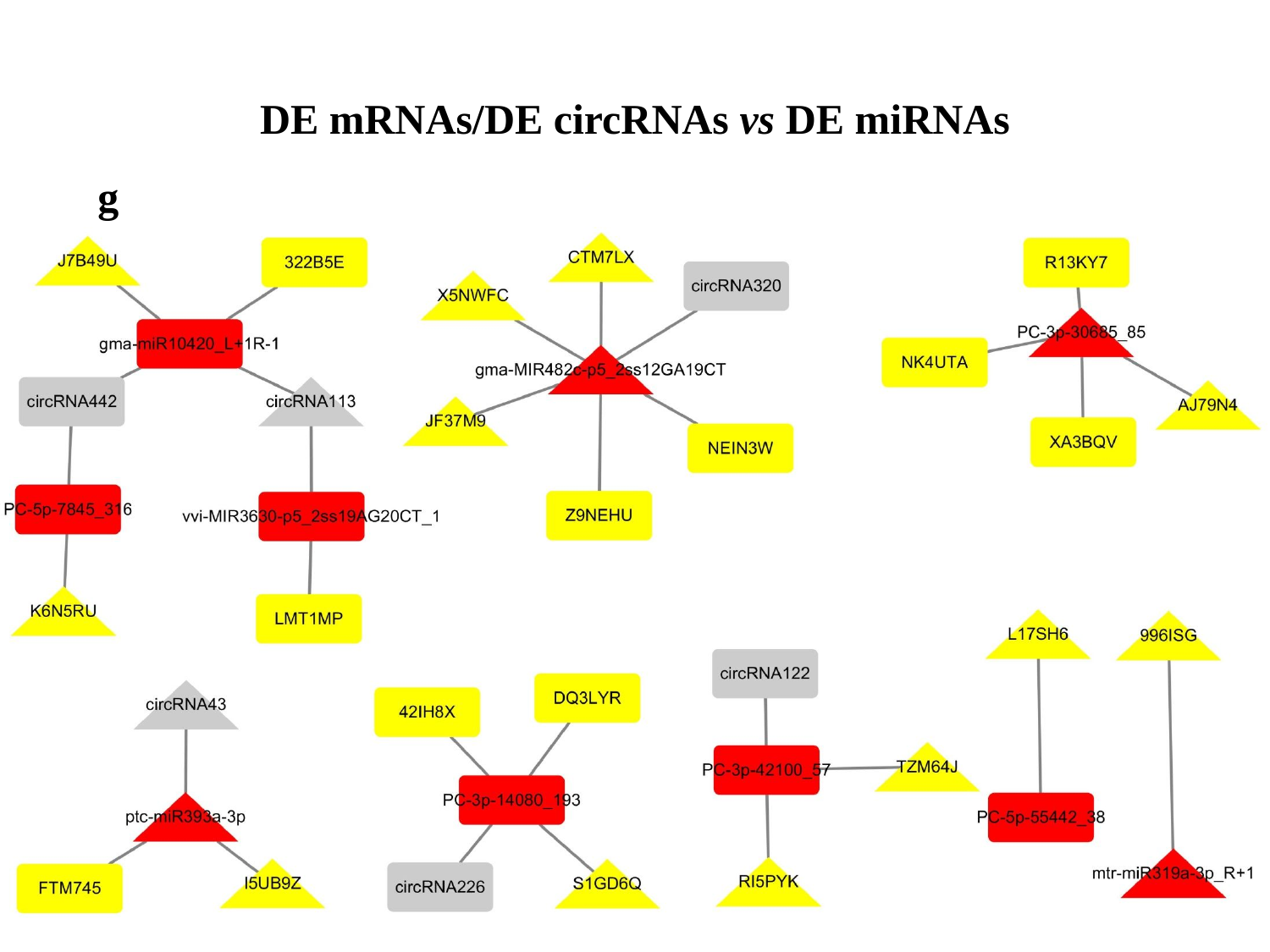

# DE mRNAs/DE circRNAs vs DE miRNAs
g

## Slide 9
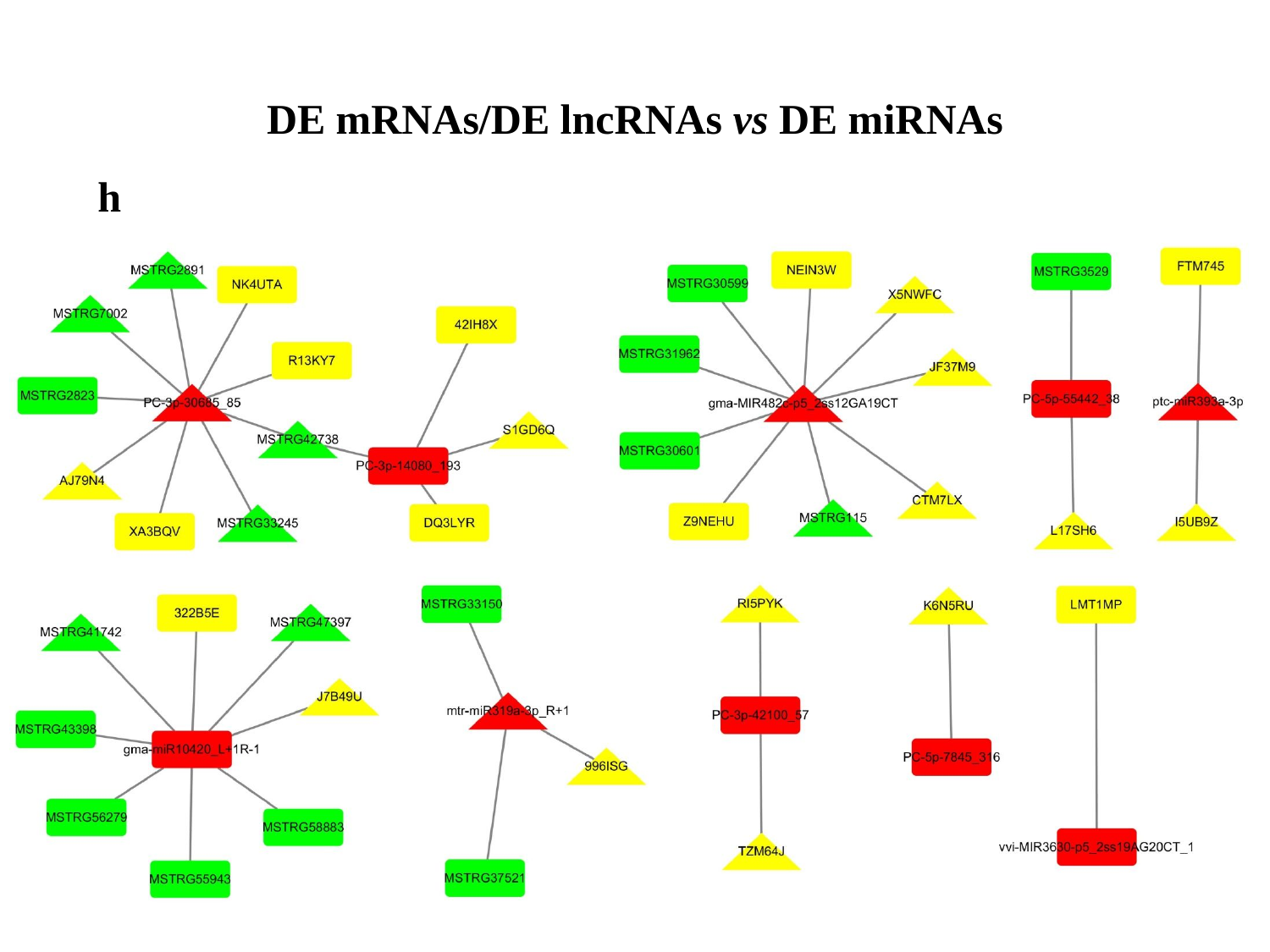

# DE mRNAs/DE lncRNAs vs DE miRNAs
h
