# Supplementary material for: Integrated analysis of the lncRNA/circRNA-miRNA-mRNA expression profiles reveals novel insights into potential mechanisms in response to root-knot nematodes in peanut
Source: BMC Genomics. 2022 Mar 28;23:239. doi: 10.1186/s12864-022-08470-3 (PMC8962500; doi:10.1186/s12864-022-08470-3)
Supplement: Supplementary file 6 — Additional file 6: Supplementary Figure 6. The differential expressed mRNAs, top GO annotation and KEGG analysis. a. The volcanic map of differential expressed mRNAs. b. The top GO annotation of differential expressed mRNAs. c. The KEGG analysis of differential expressed mRNAs. [file 12864_2022_8470_MOESM6_ESM.docx]

**Title: Integrated analysis of the lncRNA/circRNA-miRNA-mRNA expression profiles reveals novel insights into potential mechanisms in response to root-knot nematodes in peanut**

**Supplementary Figure 6. The differential expressed mRNAs, top GO annotation and KEGG analysis.** a. The volcanic map of differential expressed mRNAs. b. The top GO **a**nnotation of differential expressed mRNAs. c. The KEGG analysis of differential expressed mRNAs.
